# Autheem therapy for young Saudi infants: the whys and the impacts

**DOI:** 10.1186/s12887-025-06219-x

**Published:** 2025-10-22

**Authors:** Eman Alghaith, Jamal Ahmed Omer, Nouran Arnous, Lina Alosaimi, Fatimah Alhelal, Majdoly Alkhodair, Yossef Alnasser

**Affiliations:** 1 Department of Pediatrics, King Fahad Medical City, Riyadh, Saudi Arabia; 2 Department of Pediatrics, Dr. Soliman Fakeeh Hospital, Jeddah, Saudi Arabia; 3 College of Medicine, King Saud University, Riyadh, Saudi Arabia; 4 Pediatric Department, BronxCare Health System, 1650 Grand Concourse Ave, Bronx , New York, NY USA

**Keywords:** Saudi arabia, Infants, Development, Feeding, Traditional

## Abstract

**Background:**

In Saudi Arabia, primigravida or multigravida mothers might seek complementary medicine for their infants. When an infant struggles with colic or poor feeding, a mother will do anything to help her baby including traditional therapy. Traditional healers use a maneuver called “Autheem- عظيم” based on a common belief among the Saudi public of needing to manipulate soft palates for struggling infants.

**Methodology:**

This study is a retrospective cohort study adopted to recruit children at the age of 36 months who underwent “Autheem” therapy and those who did not as controls. Mothers in the waiting area at the general pediatrics clinics in Riyadh, Saudi Arabia were approached to answer a pre-structured survey. The “Ages and Stages Questionnaire” study tool was used to assess their children’s development.

**Results:**

The study enrolled 84 well-educated mothers (74% with college degrees in the control group and 58.5% in the study group). The main sources of information about Autheem and its indications were grandmothers and family elders. Poor feeding followed by responding to grandmothers’ pressure were the main reasons to seek this therapy. The majority of mothers (71%) admitted that if they found a solution in modern medicine, they would not have explored this therapy. Mothers noted that most healers do not wash their hands. Over one-third of mothers (36.6%) documented lethargy in the first 24 h post-therapy. One in five mothers was offered cautery therapy at the time of Autheem therapy. There was a significant difference in development between children exposed to Autheem therapy in comparison to healthy controls. Autheem exposed children were 10 times more likely to be delayed in gross motor skills. Furthermore, notable delay was documented in fine motor and problem solving despite lack of statistical significance.

**Conclusion:**

Concern about infant feeding is the main reason for seeking “Autheem.” The association of developmental delays might indicate safety concerns of this therapy on growing brains. Future advocacy should focus on elders in the family to avoid Autheem therapy until better safety data is available.

**Supplementary Information:**

The online version contains supplementary material available at 10.1186/s12887-025-06219-x.

## Introduction

Infant colic can be anxiety-provoking for many families, which might result in maternal depression, poor parent-child bonding, or inadequate breastfeeding [1]. Despite the benign nature of this problem, many families might seek alternative options to help their inconsolable infants. Those options might include traditional therapy (TT), as science has not fully understood the causes of infantile colic [2]. Even if a child does not suffer from infant colic, a mother might struggle with feeding her infant. Difficulties in breastfeeding, including poor latching, can cause distress to both growing infants and parents.

The shape of the palate, even without defect or cleft, in a newborn was thought to cause breastfeeding difficulty in scattered case reports [3, 4]. Other studies worldwide explored interventions from complementary medicine practitioners like chiropractors to treat poor latching, feeding difficulties, and infant colic [5–8]. In Saudi Arabia, traditional healers have claimed to be able to treat infant colic and poor infant feedings for decades. Saudi traditional healers use a maneuver called “Autheem”. This maneuver is based on a common belief among the Saudi public about the urgent need to elevate soft palates for struggling infants. A traditional therapist will insert their index finger into an infant’s mouth to manipulate the soft palate and elevate it until the anterior fontanelle is bulging. After elevating the soft palate, infants are expected to feed and stop crying. This raises concern about increasing intracranial pressure (ICP) as anterior fontanelle’s bulging is a sign of successful Autheem procedure. Despite the longevity of this practice in Saudi Arabia, it has not been evaluated scientifically, as far as we know. Despite all progress in pediatric care in Saudi Arabia, Autheem therapy is still unregulated. With Vision 2030, hopes for well-regulated TT, including Autheem, are high while maintaining free universal healthcare for all children. Vision 2030 is Saudi Arabia’s transformative plan to improve all infrastructure and drive the country to be a role model for healthcare in Middle East.

A newborn’s brain develops very rapidly during the first three months of life [9]. This rapid growth is susceptible to any minor insults. Those minor insults might come in shape of minor head injury or unnecessary treatment [10] . They might affect children’s development and go unnoticed without proper developmental screening. During the critical period of brain development, infants are subject to benign conditions that might expose them to unnecessary interventions. Desperate mothers might seek help for their inconsolable children who won’t stop crying or does not have satisfactory feeds. In this study, we aimed to investigate the safety and motive of seeking Autheem while assessing its effects on child development. This study can provide insights and pave the way for more research to formulate recommendations impacting early childhood experiences in Saudi Arabia and the region.

## Study methodology

### Study design

This study adopted retrospective cohort methodology. The retrospective cohort design allowed assessment of the efficacy and safety of infants’ soft palate manipulation as Saudi traditional therapy to treat infant colic and poor feeding. As mothers are the primary caregivers in Saudi Arabia, they were the main target to be surveyed in this study. Given that this is the first study to explore Autheem therapy safety, urban settings were included only at this stage. To match school readiness as a gain for participation, a development assessment at the age of 36 months was offered. Pre-structured survey was carried to understand motives and influencing factors of seeking Autheem therapy.

### Participant recruitment

Mothers were recruited from six General Pediatrics Clinics under cluster two of the Saudi Ministry of Health Primary Care Program in Riyadh, Saudi Arabia. Children were recruited to participate in this study when they presented for three-year well child visits. Mothers provided their consent before participation and were asked to complete a questionnaire that covered their demographic details, their perspective on “Autheem” therapy, and the safety measures offered upon Autheem therapy. This structured survey was built based on expert opinions, clinical observation and suggestions from two experienced Saudi pediatricians. It was tested by a pilot study on random participants for clarity and length. The survey was supplemented by a translated to Arabic and a validated tool to assess child development among both cases and control cohorts. Children who were born prematurely, diagnosed with metabolic/genetic disorders, had a history of birth asphyxia, had a history of brain injury, or had oropharyngeal abnormalities such as cleft palate were excluded from the study to minimize any confounding factors. The two groups, exposed and controls, were matched 1;1 for age, gender, baseline health, and term status of pregnancies. The match allowed balanced groups and aimed to minimize confounding as only healthy and full term children were included in both groups.

### Attitude, efficacy, and safety

The study aimed to evaluate mothers’ perceptions and satisfaction regarding Autheem therapy and their primary source of information in the first stage. Additionally, the study examined whether there were any unfavorable outcomes resulting from the therapy and whether any precautions were taken to ensure its safety. Acute adverse outcomes like hospitalization, viral infections, fractures, injuries, and lethargy were explored. Impact on development was sought out as a long-term adverse outcome of Autheem therapy. Furthermore, the influence of traditional healers on vaccine hesitancy and accessing cautery therapy were evaluated. The questionnaire was tested through a pilot study and is available in English format in the supplementary data.

### Child development assessment

To investigate the relationship between Autheem therapy and developmental milestones, a 36-month developmental assessment using a validated Arabic version of “Ages and Stages” tool was administered to both cases and healthy controls. Ages and Stages is a reliable and validated tool in Arabic and has showed good reliability and cultural sensitivity in previous [7].

### Sample size

Sample size was determined after consulting with two Saudi pediatric experts with more than 10 years of experience and extensive research background along with using an online sample size calculator (OpenEpi) to achieve a Power of 80% with 95% Confidence Interval, a minimum sample size of 38 participants per each cohort was designed to allow a statistically significant findings while avoiding type I and type II errors. Likewise, both cohorts were matched per age, gender, health status and gestational age at birth to minimize confounding and statistical errors.

### Statistics

Initially, data were logged into an Excel sheet. Then, a consulting biostatistician aided in calculating the risk ratio, odds ratio, and means using SPSS software Version 28. Utilizing z-test, a correlation between mothers’ characteristics, Autheem therapy, and developmental delays were tested using multiple logistic regression analysis.

### Ethics

Prior to data collection, this study was reviewed and obtained IRB approval from King Fahad Medical City, Riyadh, Saudi Arabia. Before surveying any mother, informed consent was discussed, and mothers were reminded of the lack of impact of participation on their clinical care. Then, a consent was obtained from patients’ parents or legal guardians prior to answering survey about children’s development and Autheem exposure. Mothers were allowed to stop or withdraw participation any time before data analysis. Cultural sensitivity and non-judgmental care was provided during data collection.

## Results

### Participants’ demographic

A total of 84 mothers who met our inclusion criteria were interviewed (43 in the control group and 41 in the exposure group). There was no variation in the gender ratio between the two groups (18 females and 23 males in the exposure group compared to 19 females and 24 males in the control group). Most mothers were between the ages of 25 and 30 years. The majority were well-educated and university graduates in both groups. Most mothers who accessed Autheem therapy for their children were multigravida mothers, and that was a determining factor in using Autheem therapy (Table 1).

**Table 1:** Demographic characteristics of study participants

Characteristic	Description	Control *N* (%)	Exposed *N* (%)	*p* Value
Number of participants		43 (100.0)	41 (100.0)	
Childs’ age (3-year)		43 (100.0)	41 (100.0)	
Childs’ gender	Female	19 (44.2)	18 (43.9)	0.979
	Male	24 (55.8)	23 (56.1)	
Mothers’ age	18–24	2 (4.7)	3 (7.3)	0.897
	25–30	18 (41.9)	19 (46.3)	
	31–40	17 (39.5)	15 (36.6)	
	> 40	6 (14.0)	4 (9.8)	
Mothers’ level of education	Intermediate level or less	3 (7.0)	3 (7.3)	0.365
	High school	7 (16.3)	13 (31.7)	
	University graduate	32 (74.4)	24 (58.5)	
	Postgraduate	1 (2.3)	1 (2.4)	
Mothers’ marital status	Married	42 (97.7)	38 (92.7)	0.42
	Divorced	1 (2.3)	2 (4.9)	
	Widow	0 (0.0)	1 (2.4)	
Number of children at time of Autheem therapy used	One‎	6 (14.0)	13 (31.7)	0.005
	Two	9 (20.9)	17 (41.5)	
	Three	12 (27.9)	6 (14.6)	
	≥ 4	16 (37.2)	5 (12.2)	
Type of feeding used before Autheem therapy	Exclusively breastfeeding	7 (16.3)	3 (7.3)	0.338
	Mixed feeding	26 (60.5)	24 (58.5)	
	Formula feeding	10 (23.3)	14 (34.1)	
Reason for Autheem therapy	Poor Feeding	35 (85)	N/A	
	Pressure from grandmothers	12 (29)	N/A	
	Infant colic	15 (36.5)	N/A	

### Reasoning and source of information

Mothers were aware and had knowledge about Autheem therapy even before their pregnancy, as it was frequently practiced in their families. The main sources of information were grandmothers and family elders. Poor feeding was the main reason to seek Autheem therapy, followed by infant colic and pressure from grandmothers (Table 1).

### Beliefs and attitudes toward autheem therapy

Despite high beliefs in Autheem therapy (81%), more than two-thirds of mothers (71%) expressed hesitancy in using TT if solutions were provided by modern medicine. However, satisfaction with Autheem therapy was high among the exposure group (83%). Strong belief and satisfaction were paired with the desire to offer Autheem therapy to their next child among the exposure group. Additionally, most mothers would advocate to use Autheem for poor feeding rather than infant colic (78% vs. 46.3%, respectively).

### Safety and hygiene

**Fig. 1:**
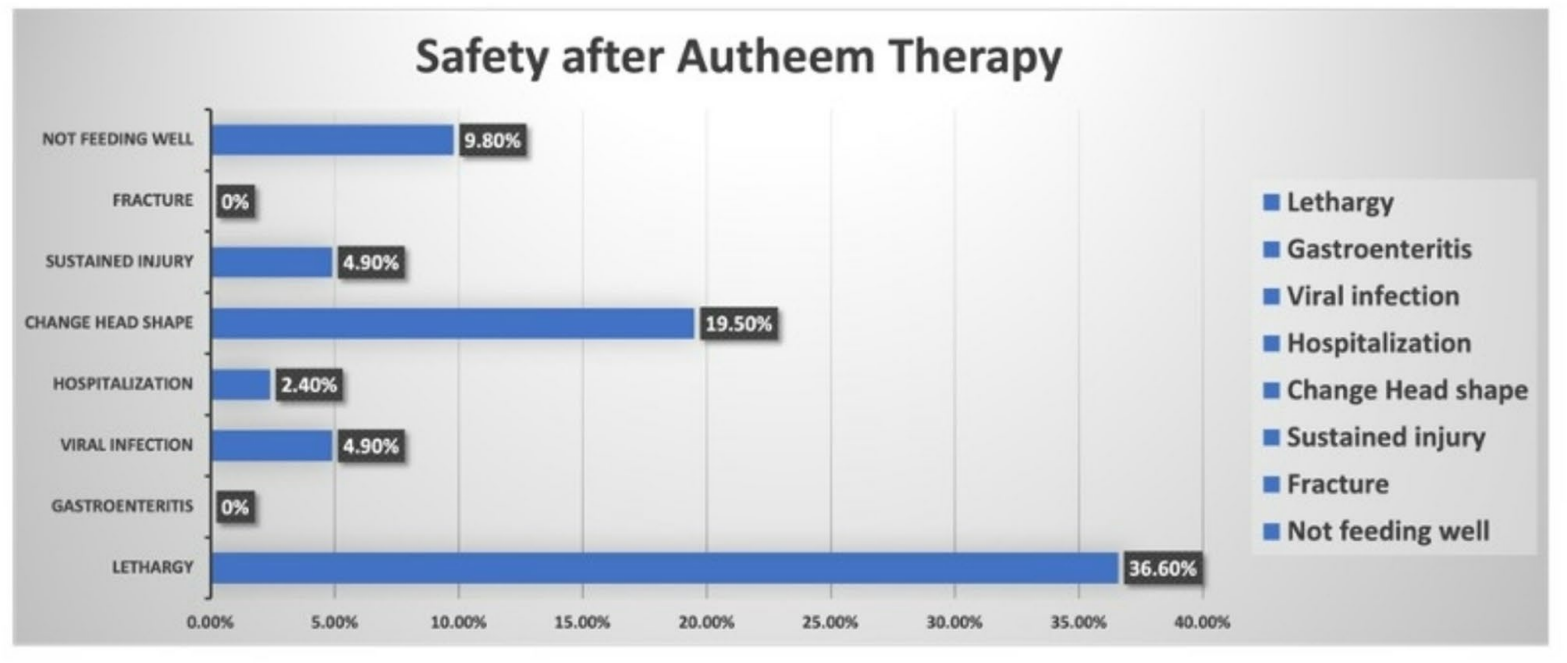
Safety after Autheem therapy

Half of the mothers from the exposure group noticed that traditional healers did not wear gloves prior to manipulating their babies’ soft palates. Despite the lack of reported major side effects after therapy and only one hospitalization, one-third of mothers in the exposure group documented lethargy in the first 24 h post-therapy **(**Fig. 1**).** One in five mothers in the exposure group was offered cautery therapy at the time of Autheem therapy. There was no impact of Autheem therapy on vaccine schedules or vaccine hesitancy.

### “Autheem” therapy impact on child development

**Fig. 2:**
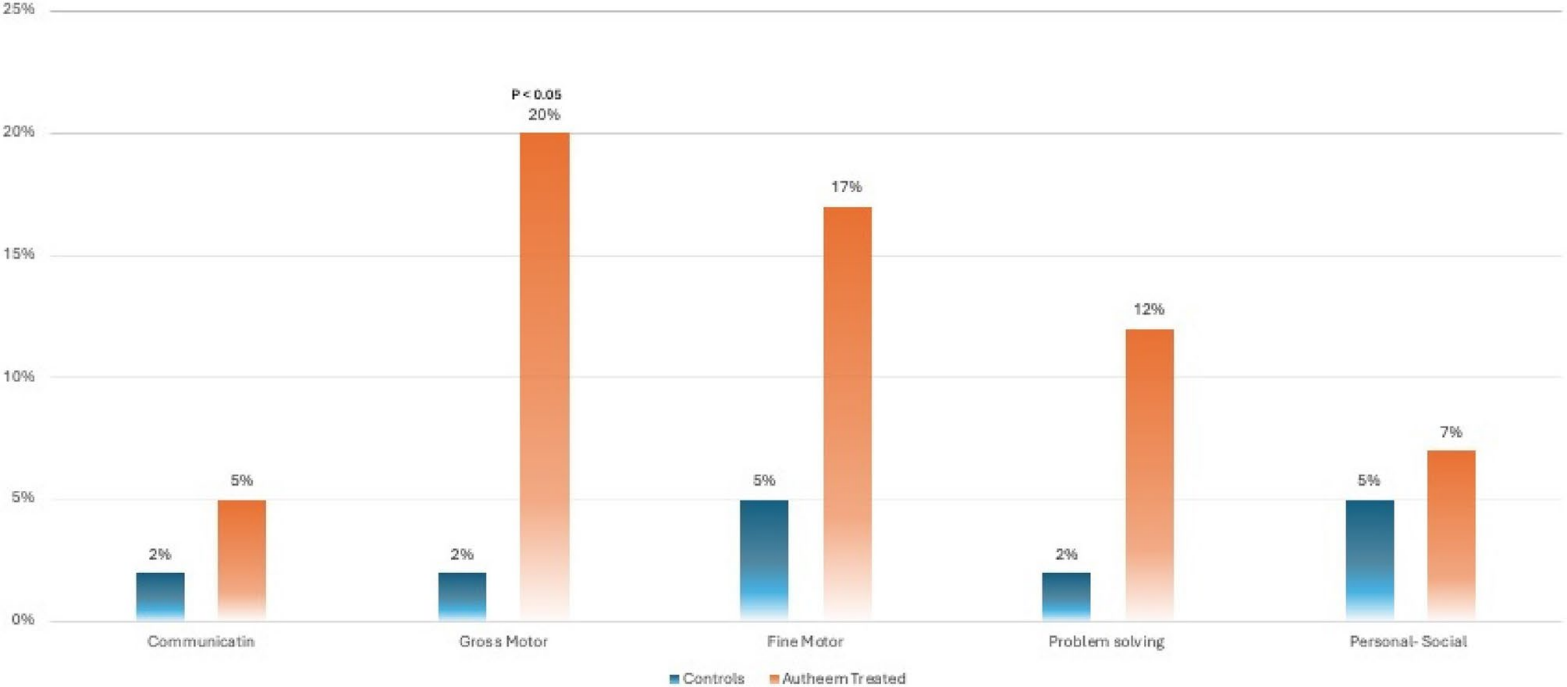
Areas of developmental delay on ages and stages in children exposed to “Autheem” therapy

Ages and Stages Questionnaire revealed a significant difference in development between children exposed to Autheem Therapy compared to healthy controls. Autheem-exposed children were 10 times more likely to be delayed in gross motor skills in comparison to non-exposed children (OR 10.18). Using the student z-test, the delay in gross motor skills was statistically significant with a p-value of 0.009. Children exposed to Autheem Therapy were found to be delayed in Fine Motor (7 cases, 17%) and problem-solving (5 cases, 12%) with a p-value of (0.08 vs. 0.07, respectively) **(**Fig. 2**).**

## Discussion

In Saudi Arabia, people have sought TT over the years and trust healers’ care [11]. Despite advancements in modern medicine and improvement of the child healthcare system in the country, many families might consider TT for their children [11]. The interest in TT was documented by AL-Rowais et al. to be higher in suburban and rural areas compared to the capital city [12]. Furthermore, TT was sought by many Saudi public as the last resort when modern medicine is inaccessible or fails to address their concerns [10, 11]. This was parallel to our findings as many mothers admitted a lack of interest in Autheem therapy if they found a solution to poor feeding in modern medicine. However, certain TT practices are deeply embodied in Saudi culture and religious beliefs [12]. Infants can be subject to cauterization, offered to drink holy water or apply a paste of traditional herbs on their anterior fontanelle to help with infant colic and poor feeding. With rising numbers of chronic non-communicable diseases like diabetes and hypertension in Saudi Arabia with no cure in modern medicine, TT might gain more popularity after the COVID-19 pandemic and the trust issues that came with it [13, 14]. Our study raises a red flag about link of Autheem therapy to development delays and ask for more research and further scrutiny of this long traditional practice. To ensure Saudi public safety, especially for children, TT should be integrated with modern medicine and studied further. The call for integration is not novel and has been advocated for years by medical and public health champions in the young nation [15].

Our small sample size can be explained by the hesitation of mothers to disclose their access to TT with their healthcare providers. This was found on other studies when most caregivers utilizing TT for children had not disclosed them to their physicians [16, 17]. The reasoning for not disclosing is not clear and has not been explored before. It could be fear of judgment, lack of trust, or concern about being reported to child protection teams. Still, these are just speculations. Until better data come along, Saudi pediatricians need to communicate clearly and establish an open, non-judgmental relationship with parents.

Grandparents and families’ elders are huge influencers for seeking TT [18]. This was noted in our study, as pressure from grandmothers was one of the main reasons for seeking “Autheem” therapy. Despite the high educational level of mothers in our study, grandmothers were the main source of information for “Autheem” therapy and TT, which is similar to previous reports [16]. However, our finding deviated from another Saudi study’s finding of the popularity of TT among illiterate mothers over a decade ago [10]. Today, Saudi women are empowered and well educated, which was reflected in our findings. Unfortunately, a high level of education does not equate to high health literacy. Poor health literacy is well documented among the Saudi population [17]. To promote better health for all children in Saudi Arabia, investment in promoting health literacy among parents is highly needed.

Another example of inadequate health literacy was found when mothers were asked about infant colic in one study. The majority of mothers had no previous understanding of the condition. In the same study, information obtained from healthcare providers about infant colic was the least common source of information [19]. Instead of just blaming mothers, Saudi healthcare providers should prioritize anticipatory guidance and health promotion in regular follow-up visits during well-baby clinics. A good understanding of feeding expectations and infant colic might help in preventing unnecessary visits for Autheem therapy.

There is little data to support the safety and efficacy of TT, including Autheem therapy, and further research is required to identify any potential risks [20]. In one literature review, three deaths were reported in children treated with a manual therapist [21]. In our study, we found that one of three infants who underwent Autheem therapy developed lethargy in the first 24 h post-therapy. Lethargy raises concern about any potential neurologic insult to the growing brain. Insults at such a vulnerable time for growth and development can hinder the acquisition of typical development milestones. Among infants receiving Autheem therapy, gross motor delay was significantly impacted. This therapy altered fine motor skills and problem-solving despite a lack of statistical significance. These alarming trends in fine motor skills and problem-solving can guide future research and might be detected with higher power. Additionally, there was one hospitalization post-therapy. This indicates a number needed to harm is 1:40. This is a significant number that can put 2.4% of children accessing this therapy at risk of unnecessary hospitalization. At a public health level, this will drive health costs higher while lowering children’s quality of life [22–24].

Our study has several limitations, including the relatively small sample size. This could be due to several factors, including the limited study geographic area. Moreover, there were no specific records of infants who underwent “Autheem” therapy, which could have made it easier for the research group to reach a large study population. We assessed children’s developmental milestones at 36 months of age, but we did not follow them at different ages to evaluate the possibility of the long-term impact of this therapy on their development. Furthermore, our study type is subjective to recall bias and did not specify timing of Autheem therapy on the growing brain. Despite all limitations, this study helped in establishing an association between this long culturally accepted therapy and developmental delay to set an alarm for more research to explore causality. Another strengths of this studies is its novelty and ability to investigate an original and culturally acceptable practice in Saudi Arabia and provide primary data about reasoning and safety of such practice.

## Conclusions

Autheem therapy is a culturally sensitive therapy with long-lasting practice in Saudi Arabia. The association of developmental delays sparks safety concerns about this therapy on the growing brains of children. Further larger and prospective studies are needed to determine the safety of this traditional therapy. This will guide Saudi child health practitioners in advocating for families and young infants.

## Supplementary Information

Supplementary Material 1.

## Data Availability

All data can be made available upon request from the primary investigator.
