# Supplementary material for: Autheem therapy for young Saudi infants: the whys and the impacts
Source: BMC Pediatr. 2025 Oct 22;25:848. doi: 10.1186/s12887-025-06219-x (PMC12542447; doi:10.1186/s12887-025-06219-x)
Supplement: Supplementary file 1 — Supplementary Material 1. [file 12887_2025_6219_MOESM1_ESM.docx]

**Questionnaire about Autheem Complementary Therapy**

We are a research team from a group of pediatricians and medical students, led by the principal investigator Dr. Yossef Al-Nasser. We aim to study the popular treatment of “Autheem” in Saudi society. The study aims to find out if your child has ever been exposed to “Autheem” treatment. We hope you to participate to understand this treatment and what are the mothers' motives for obtaining it. We also aim to understand to what extent is it safe and does it carry the risk of any infections or infectious diseases. Then we will evaluate your child's development and share the results with you if you wish. If you agree to participate and your child was previously exposed to “Autheem” treatment and he/she is now three years old, I hope you will complete the following questionnaire

1. **Mothers’ age.** ( )18-24. ( )25-30. ( ) 31-40. ( ) above 40
2. **Mothers’ level of education.** ( )Intermediate level or less. ( ) High school. ( ) University graduate. ( ) postgraduate
3. **Mothers’ marital status.** ( ) married. ( ) divorced. ( ) widow
4. **Number of children at time of Autheem therapy used.** ( ) 1. ( ) 2. ( ) 3. ( ) 4 or more
5. **Type of feeding used before Autheem therapy.** ( ) Exclusively breastfeeding. ( ) breastfeeding with formula feeding. ( ) formula feeding
6. **When did you hear about Autheem therapy.** ( ) before pregnancy and it is commonly used in our family. ( ) While I was pregnant. ( ) Immediately after giving birth in the first weeks

( ) when my child 2 months of age or more

1. **Your source of information about Autheem therapy from.** ( ) Your mother or senior females in the family. ( ) friends from your same generation. ( ) social media “Instagram, snapchat and whatsapp”. ( ) traditional media “Television, broadcast and newspapers”
2. **You used Autheem therapy to treat your child from.** ( ) Infant colic. ( ) poor feeding. ( ) as a result to the pressure from senior females in your family. ( ) low weight or low weight gain

**Please Answer the following questions at a scale from 1-5, In which:**

1: Strongly agree, 2: Agree, 3: Neutral, 4: Disagree, 5:Strogly disagree

| If my child had improved with modern medicine, I wouldn't have use Autheem therapy | - Strongly agree | - Agree | - Neutral | - Disagree | - Strongly disagree |
| --- | --- | --- | --- | --- | --- |
| I believe in Autheem Therapy | - Strongly agree | - Agree | - Neutral | - Disagree | - Strongly disagree |
| There is no cure of Autheem in modern medicine | - Strongly agree | - Agree | - Neutral | - Disagree | - Strongly disagree |
| My next child with undergo Autheem therapy if he/she needed it | - Strongly agree | - Agree | - Neutral | - Disagree | - Strongly disagree |
| I advise other mothers to perform Autheem therapy for poor breastfeeding. | - Strongly agree | - Agree | - Neutral | - Disagree | - Strongly disagree |
| I advice other mothers to perform Autheem therapy for infant colic | - Strongly agree | - Agree | - Neutral | - Disagree | - Strongly disagree |
| My child improved after Autheem therapy | - Strongly agree | - Agree | - Neutral | - Disagree | - Strongly disagree |
| Traditional healer washed his/her hand before applying Autheem to my child | - Strongly agree | - Agree | - Neutral | - Disagree | - Strongly disagree |
| Traditional healer wore gloves before applying Autheem to my child | - Strongly agree | - Agree | - Neutral | - Disagree | - Strongly disagree |
| My child had gastroenteritis after Autheem Therapy | - Strongly agree | - Agree | - Neutral | - Disagree | - Strongly disagree |
| My child had viral infection after Autheem Therapy | - Strongly agree | - Agree | - Neutral | - Disagree | - Strongly disagree |
| My child needed hospitalization week after Autheem therapy | - Strongly agree | - Agree | - Neutral | - Disagree | - Strongly disagree |
| My child head shape changed after Autheem therapy | - Strongly agree | - Agree | - Neutral | - Disagree | - Strongly disagree |
| My child sustained an injury to his palate after Autheem therapy | - Strongly agree | - Agree | - Neutral | - Disagree | - Strongly disagree |
| My child had fractures after Autheem Therapy | - Strongly agree | - Agree | - Neutral | - Disagree | - Strongly disagree |
| My child couldn’t feed well after Autheem therapy | - Strongly agree | - Agree | - Neutral | - Disagree | - Strongly disagree |
| My child became sleepier and more lethargic after Autheem Therapy | - Strongly agree | - Agree | - Neutral | - Disagree | - Strongly disagree |
| Traditional healer who applied Autheem Therapy, recommended me to leave modern medicine to treat my child. | - Strongly agree | - Agree | - Neutral | - Disagree | - Strongly disagree |
| Traditional healer who applied Autheem Therapy, recommended me not to vaccinate my child | - Strongly agree | - Agree | - Neutral | - Disagree | - Strongly disagree |
| Traditional healer who applied Autheem Therapy, recommended me to iron my child | - Strongly agree | - Agree | - Neutral | - Disagree | - Strongly disagree |

Arabic version of Ages and stages of 36 months will be given next.
